# Mental health literacy and borderline personality disorder (BPD): what do the public “make” of those with BPD?

**DOI:** 10.1007/s00127-014-0936-7

**Published:** 2014-07-27

**Authors:** Adrian Furnham, Vanessa Lee, Vladimir Kolzeev

**Affiliations:** 1 Research Department of Clinical, Educational and Health Psychology, University College London, London, UK; 2 Norwegian Business School (BI), Nydalsveien, Oslo, Norway

**Keywords:** Mental health literacy, Borderline personality disorder lay beliefs, Mental health disorders, Help recommendations

## Abstract

**Introduction:**

This study was part of a programmatic series into mental health literacy, specifically lay people’s knowledge of causes, manifestations and cures of Borderline Personality Disorder (BPD). The aim was to determine to what extent non-experts understood BDP and to what extent they held erroneous beliefs about specific aspects of the causes and cures for the disorder

**Method:**

A convenience sample of 102 participants completed a vignette-identification task that required them to answer open-ended questions about hypothetical people with four psychological problems, one of which was BPD and a 50-item questionnaire divided into three sections about BDP.

**Results:**

Analysis of the vignette identification task revealed very low rates of recognition for BPD, with participants significantly more likely to identify depression, schizophrenia or psychopathy. Participants’ generally favoured psychological and sociological treatments, as well as rating early trauma and stress as possible causes of the symptoms of the person described in the BDP vignette. There were logical relationships between perceptions of cause and cure.

**Conclusion:**

The results suggest that participants hold certain coherent beliefs (psychological, sociological, biological or theological) regarding aetiology and treatments of BPD. Further, the findings suggest the need for greater awareness and educational programmes to inform the general public accurately regarding BPD and to improve mental health literacy.

## Introduction

Mental health literacy (MHL) refers to people’s knowledge, as well as beliefs, about the diagnosis and treatment of mental illness. A considerable amount of research has been done in the area of MHL initiated by Jorm and others [20]. Various recent reviews have appeared attesting to the growth of the field [18].

Each component of MHL has attracted a great deal of interest and attention from researchers in social, as well as health, psychology over the last decade [3, 10, 13, 20, 21, 23, 30].

There have been studies comparing people from different communities, countries, and professions [27], and most have concentrated on depression and schizophrenia using vignette methodology [4].

This paper is concerned with MHL, particularly with respect to borderline personality disorder (BPD). Lay people, in the context of this study, are defined as members of the general public who hold no professional qualifications in the fields of Psychology or Psychiatry. In our study, we did include some participants who had some education in either psychology or psychiatry and looked specifically at those effects. Most MHL studies have investigated the knowledge of adult members of the general public, though many have concentrated on specific groups like students, relatives, or patients themselves [11, 14, 18].

Typically, vignette identification methodology is used in studies of public MHL, where participants are provided with vignettes describing characters, which they have to label, though there are recognized problems with this technique [28]. Different vignettes have tended to yield different responses in part because of their details and length and partly because of issues concerning comorbidity.

### The recognition of different disorders

A wide variety of disorders have been considered in the MHL literature, with depression and schizophrenia being the most widely and frequently investigated [18, 20]. One methodological issue concerns whether participants use the official label, which is regarded as the only “correct” answer, or whether from their responses it is apparent they have a good understanding of the problem but do not know the official diagnostic term for it. So as to make this point we have put the term “correct” in italics.

Fewer studies have looked at the personality disorders (PDs). More recent studies extended to test the MHL of other disorders and compared recognition rates among different disorders [7, 19]. For instance, in a cross-cultural study conducted by Loo et al. [24] in the United Kingdom, Hong Kong, and Malaysia, use of the ‘correct’ identification rates of nine different mental disorders were compared with each other. For British participants, mental disorders with the highest ‘correct’ labeling rates were OCD (75 %) and depression (70 %), whereas the lowest were bipolar disorders (18 %) and social phobia (2 %). A similar study has emerged from mainland China [15].

A few studies have looked at the identification of BPD; Furnham et al. [9] tested 187 adults on their ability to recognize 10 PDs and found that BPD was the least well identified and judged as least adjusted. Furnham and Winceslaus [12] tested 223 adults and found only 6.3 % used the term BPD and that 44.4 % thought they were either depressed or bipolar, and nearly a third said they had no idea. Of all the 10 PD vignettes, the hypothetical person with BPD was judged as least happy and second least successful at work, and having good personal relationships.

It seems that certain disorders, such as depression, are studied and discussed much more frequently than others, such as bipolar disorder and social phobia, partially due to media coverage and prevalence of diagnosis. As a consequence, it may be expected that some are more easily identified whereas some are nearly always mislabeled, which in turn raises concerns about reliability of diagnosis as well as efficacy of treatment seeking [5].

One of the “big issues” for PD researchers and practitioners is the issue of comorbidity. There is considerable evidence of comorbidity of the PDs with a wide range of Axis 1 disorders [25]. This may in part account for different results from different studies which used somewhat different vignettes, either because they emphasized or omitted different features, or else gave hints of comorbidity.

The Lenzenweger et al. [25] analysis suggested that BPD seemed particularly prone to comorbidity. In this study, one of the first in the area, we examined MHL of BPD patients’ comorbid with four other common problems. Out question is what effect this had on the recognition of BPD as well as the other disorder and the consequent reaction to the patients.

### This study

This study focuses on BPD. Very few studies have looked at non-experts views on this disorder although some studies have looked as such things at how nursing staff react to patients with the label of BPD [26]. BPD is a cluster two personality disorder (DSM-IV, DSM-V) [1, 2] characterized by emotional instability, impulsivity, disturbed cognition, and intense unstable relationships. It has a prevalence of 1–2 % in the general population, the figure rising to 10–20 % in patient populations (DSM-IV, 2000). A recent large scale America study found lifetime BPD prevalence rates of 5.9 % but no difference between men and women [16]. That study also found a high co-occurrence with anxiety and mood disorders as well as bipolar, narcissistic, and schizotypal disorder.

The issue of comorbidity and its impact on reliable diagnosis and the “inflation of the mental disorders” have been consistently discussed and appear to be particularly relevant to BPD [6, 8].

For a formal diagnosis to be made, individuals must meet five of the nine criteria. As with many mental illnesses, the causes of BPD are complex and multifactorial, including trauma, family chaos, disrupted attachments, multiple caregivers, parental neglect, alcoholism, and affective instability among the family members (DSM-IV, 2000). It is also recognized that there is evidence of a genetic component and clear evidence of biological factors in BPD [22].

Given the high comorbidity associated with BPD, some practitioners have argued against its classification as a unique, distinguishable disorder. A large body of research has demonstrated that BPD overlaps with several other personality disorders; that is, that the incidence of comorbidity is very high [6, 16].

This study set out to investigate three issues and test three hypotheses. Firstly, whether BPD can be as easily recognized as mental disorders (depression and schizophrenia). The first hypothesis (H1) predicted that more participants would successfully identify depression and schizophrenia (individually) than BPD. This is both because of the higher frequency, certainly of depression and schizophrenia in the population, but also because the labels are better known and more widely discussed in the popular press. The second hypothesis is that BPD would be seen as less distressing and dishabilitating than either depression and schizophrenia, and that persons with BPD would be less in need to seek professional help (H2). The third hypothesis predicted that participants with some formal training in psychology, medicine, or psychiatry would be better able to recognize the mental disorders in general, and BPD in particular (H3).

## Method

### Participants

A total of 193 participants have taken part in the study. Sampling was opportunistic and participation was voluntary, with no remuneration provided. The age range was from 18 to 62 years (*M* 26.08, SD 9.82); 113 were male and 80 female; the majority of the sample were of White British ethnicity (46.1 %), followed by Asian (40.4 %), Mixed (7.9 %) and Black (5.6 %); as for education, 1.1 % achieved GCSE or equivalent, 37 % A-levels or equivalent, 38.1 % Undergraduate degree and 23.8 % Postgraduate degree (Masters or Doctorate); 34.7 % of the sample reported formally studied psychology to some level; and 19.7 % had been personally treated for a psychological disorder.

### Materials

There were eight vignettes in the questionnaire: six BPD, one depression, and one schizophrenia all of which conformed to the criteria of DSM-IV. The BPD vignettes were sourced from a textbook by Gunderson [17] and the other two were taken from Spitzer et al. [29]. The vignettes were around 100 words in length. They were taken from the second chapter of the book that dealt with, and explained in detail, the issue of comorbidity and differential diagnosis. Gender was kept from the original source with (4 females, 4 males). The questionnaire was piloted in regards to ease of understanding both the vignettes and the questions. It is also worth noting that the BPD vignettes (in keeping with epidemiology studies) had a comorbid factor also mentioned in the source textbook, two were type II bipolar depression, two post-traumatic stress disorder (PTSD), one narcissistic personality disorder (NPD), and one antisocial personality disorder (ASPD)/substance abuse. An example (BPD/PTSD) can be seen below:

Tanya, a 44-year-old woman presented with flashbacks that disrupted her sleep and concentration. Her childhood included eight hospitalisations between ages 13 and 18 for treatment of a congenital disease. Twenty-six years later, she could still access the feeling of being “helpless and alone”. In response, she would become agitated, with bursts of accusatory, offensive anger toward her husband and children, which she would then deeply regret as unfair. This remorse then prompted self-destructive or suicidal impulses.

The open-ended question was “What, if anything, would you say is X’s main problem?” The character adjustment rating part of the questionnaire included questions regarding: how distressing the disorder is, difficulty of treatment, amount of sympathy the participant would feel toward the person, happiness of the person described, their work success and how satisfying their personal relationships are. The help recommendation sections of the questionnaire included a question regarding whether the participant would suggest the person described seeks help for the problem. The following options were then provided: none, friends, parent, other family members, GP, psychologist/psychiatrist, books and internet; the likelihood of choosing a particular place was asked to be provided. All responses have been measured on a 1–7 Likert scale with 1 being “Not at all/Not very likely” and 7 being “Definitely/very likely” for character adjustment and help recommendations, respectively. These are shown in detail in “Appendix”.

### Procedure

Prior to commencement the appropriate ethical committee approved the study. Data was collected by researchers approaching members of the general public in central London and on two university campuses. It took around 20 min to complete the questionnaire. Where possible, participants were debriefed.

## Results

The first part of the analysis was concerned with coding the content of the open-ended questions. A simple framework was developed in which in order for a response to be classified as “correct” the answer for BPD had to be either “borderline personality (disorder)” or “personality disorder”, since only one was investigated further clarification was not required. For depression the only “correct” response was “depression” since other labels such as “lack of motivation” could actually be considered symptoms rather than the disorder correctly identified. Finally for paranoid schizophrenia accepted terms were “paranoid/paranoia”, “schizophrenia”, and “psychosis”. Responses were coded as dichotomous variables for all eight vignettes. The frequencies of “correct” identification were calculated using Cochran’s *Q* which proved significant (*Q* (7) = 834.33, *p* < 0.001), demonstrating the presence of significant differences within the vignettes. Pairwise comparisons with Bonferroni correction were carried out post hoc to determine the exact differences between different from each other (*p* > 0.05).

During the initial coding stage, it became apparent that a number of responses provided were identifying the comorbid disorders associated with the BPD vignettes. To investigate this further the open-ended question has been recoded into a new set of dichotomous variables, which treated comorbid disorder identification as “correct”. The frequencies were calculated and another Cochran’s *Q* test conducted to identify significant differences within the six BPD vignettes in terms of the comorbidity. This was significant *Q* (5) = 142.728, *p* < 0.001. Bonferroni-corrected post hoc pairwise comparisons have then been carried out to determine the exact differences between the rates of recognition of comorbid disorders.

Table 1 shows that most participants either gave a diagnosis with a symptom of a disorder, or named an unrelated disorder, often from Axis 1 or quite unrelated to the vignette at all. The pattern did differ between the different BPD vignettes.

**Table 1:** Responses for the different disorders

	BPD 1	BPD 2	BPD 3	BPD 4	BPD 5	BPD 6	Depr	Schiz
Correct	4.1	4.1	0.5	2.1	1.0	2.1	72.5	65.8
Another disorder	16.1	22.8	28.0	8.8	3.1	33.7	–	–
Psych symptom	62.7	43.5	59.6	68.9	65.4	36.7	7.3	7.3
Unrelated	15.0	28.0	11.9	19.7	29.5	27.5	20.2	26.9
No issue	2.1	1.6	–	0.5	1.0	–	–	–

This analysis revealed significant variation in how the vignettes were perceived despite all six of them clinically belonging to BPD. Since the focus of this study was aimed at comparing BPD to better-recognized disorders, the variation introduced by comorbidity could be a potential cofounding variable. To, at least partially, negate its effect on the internal validity of the study the BPD vignettes have been averaged for the following analyses.

### Vignette character adjustment

This part of the analysis investigated the scores given for various aspects of living with a psychological disorder (see “Appendix”, questions 2–7). All analyses were significant: level of distress—*F*(1.802, 335.225) = 70.953, *p* < 0.001; difficulty of treatment––*F*(1.326, 246.583) = 19.417, *p* < 0.001; level of sympathy—*F*(1.900, 353.310) = 39.875, *p* < 0.001; level of happiness—*F*(2, 370) = 24,756, *p* < 0.001; success at work—*F*(1.899, 347.505) = 73.622, *p* < 0.001; satisfaction in personal relationships—*F*(1.840, 342.288) = 12.821, *p* < 0.001.

Post hoc pairwise comparisons with Bonferroni correction were then carried out to determine the exact conditions that were significantly different from each other. As can be seen from Table 2, BPD was considered significantly: less distressing than both schizophrenia and depression; harder to treat than depression, but easier to treat than schizophrenia; received the lowest amount of sympathy; the highest level of happiness; highest success at work; and best interpersonal relationship quality.

**Table 2:** Ratings of vignette character adjustment (mean and SD)

Mental disorder	Distress	Difficulty	Sympathy	Happiness	Success at work	Personal relationships
BPD	**5.40** (0.82)	**4.63** (1.16)	**4.63** (1.16)	2.42 (0.67)	3.29 (0.77)	2.57 (0.77)
Depression	**5.74** (1.42)	**4.12** (1.73)	**5.28** (1.62)	1.82 (1.20)	2.24^a^ (1.31)	2.25^a^ (1.71)
Schizophrenia	**6.39** (1.05)	**5.65** (3.91)	**5.79** (1.35)	2.06 (1.23)	2.19^a^ (1.36)	2.10^a^ (1.25)

Superscripted means (a) in each column are not significantly different from each other (*p* > 0.05)

Ratings for BPD were averaged from the first six vignettes; means in bold are greater than half of the rating scale (over 4)

*BPD* borderline personality disorder

### Help recommendations analysis

This analysis focused on questions 8 and 9. Results showed differences in reaction to the vignettes and each was significant: mean likelihood of suggesting help—*F*(2, 368) = 43.581, *p* < 0.001; likelihood of coping alone—*F*(2, 356) = 10.074, *p* < 0.001; likelihood of friends helping—*F*(1.733, 318.802) = 41.050, *p* < 0.001; likelihood of parents helping—*F*(1.855, 344.977) = 26.696, *p* < 0.001; likelihood of other family members helping—*F*(2, 364) = 33.355, *p* < 0.001; likelihood of a GP helping—*F*(1.863, 337.138) = 18.786, *p* < 0.001; likelihood of a psychologist/psychiatrist helping—*F*(1.593, 297.884) = 16.553, *p* < 0.001; likelihood of books helping—*F*(1.869, 347.712) = 13.929, *p* < 0.001; and likelihood of the internet helping—*F*(1.845, 343.104) = 17.635, *p* < 0.001. To determine which particular vignettes were significantly different from each other, Bonferroni-corrected post hoc pairwise comparisons were also conducted.

Table 3 demonstrates that in regards to BPD participants were significantly less likely to suggest seeing help than for either depression and schizophrenia; coping on one’s own was suggested more than with schizophrenia; friends were suggested more than with schizophrenia, but less than with depression; parents were suggested less than with depression; family members were also considered as a more unlikely source of help than with depression; GP was recommended less than for both depression and schizophrenia; psychologist/psychiatrist was recommended less than for schizophrenia; books were recommended more than for schizophrenia, but less than for depression; and the internet was also recommended more than for schizophrenia, but less than for depression.

**Table 3:** Ratings of help recommendations

Mental disorder	Mean likelihood of suggesting help (SD)	None: able to cope alone (SD)	Friends (SD)	Parents (SD)	Family (SD)	General practitioner (SD)	Psychologist (SD)	Books (SD)	Internet (SD)
BPD	**5.81** (0.85)	1.81^a^ (1.04)	**4.70** (1.45)	**4.34** ^a^ (1.41)	3.97^a^ (1.41)	**4.65** (1.59)	**5.82** ^a^ (1.08)	3.55 (1.63)	3.03 (1.59)
Depression	**6.30** (1.24)	1.63^a^ (1.35)	**5.32** (1.87)	**5.19** (1.94)	**4.86** (2.00)	**5.28** ^a^ (2.00)	**6.01** ^a^ (1.68)	3.89 (2.19)	3.48 (2.19)
Schizophrenia	**6.55** (1.01)	1.40 (1.21)	**4.11** (2.27)	**4.44** ^a^ (2.24)	3.39^a^ (2.24)	**5.36** ^a^ (2.21)	**6.41** (1.38)	3.20 (2.28)	2.73 (2.06)

Superscripted means (a) in each column are not significantly different from each other (*p* > 0.05)

Ratings for BPD were averaged from the first six vignettes; means in bold are greater than half of the rating scale (over 4)

*BPD* borderline personality disorder

### Personal history analysis

To explore the relationship between demographic factors and disorder identification several bivariate analyses have been carried out. The first set of variables included formal psychological education and disorder identification accuracy: a weak, but significant positive correlation was obtained, *r* = 0.31, *p* < 0.001. Better educated people had higher MHL. Secondly gender was tested against recognition of disorders to investigate gender differences in MHL: a weak, but significant positive correlation was obtained, *r* = 0.13, *p* < 0.05 which indicated that females had higher MHL than males. Thirdly, personal experience of psychological treatment was weakly, but significantly and positively correlated with identification accuracy, *r* = 0.17, *p* < 0.05. Lastly, personal experience of psychological treatment was correlated with the overall (average across all vignettes) measure of likelihood of recommendation to see a psychologist/psychiatrist: a significant negative correlation has been found, *r* = −0.14, *p* = 0.05.

## Discussion

This study made numerous predictions all of which have to be addressed. The first prediction (H1) regarding the recognition was confirmed. Participants were much better at recognizing depression and schizophrenia compared to BPD. Indeed recognition rates for BPD varied from 0.5 to 4.1 % with an average of 2.3 %, which is both under 10 % and is in keeping with existing research: Furnham et al. [9] who found 1 %; Furnham and Winceslaus [12] who found 6.3 %. Additionally pairwise comparisons have revealed that there were no significant differences within the BPD vignettes, with all of them being significantly different from the ‘common’ disorders. Interestingly recognition rates in this study were similar to the prevalence rates of BPD in the general population [16].

The prediction of the majority of the sample correctly labeling depression and schizophrenia was also confirmed with 72.5 and 65.8 %, respectively. These rates are similar to existing findings such as 97 and 61 % of Furnham et al. [10], making it possible to suggest that these vignettes were an appropriate baseline against which BPD could be compared. They were also not significantly different from each other.

Another prediction was concerned with differences between the ratings provided for BPD vignettes in comparison to depression and schizophrenia within the “adjustment” section. Significant differences were found for each single item with examples including BPD yielding lowest sympathy rating, highest work success and interpersonal relationships quality, and higher difficulty of treatment than depression. Participants were clearly not sympathetic to the BPD “patients” in the vignettes who did not provoke sympathy, appeared harder to be helped and was not seen as an interference with everyday life; unlike depression, which seemed to be treated as a crisis rather than a constant state. It should, however, be mentioned, that for both work success and relationship quality despite being “the highest”, this is relative to the other two mental disorders with numbers for BPD still being low and suggesting dysfunction.

As for help recommendations, H2 was also confirmed: BPD being associated with the lowest likelihood of suggesting seeking help, highest “coping alone” and lowest GP and Psychologist/Psychiatrist recommendations.

It was also expected that some formal psychological education, albeit unspecified in amount and depth, would positively affect disorder recognition. A significant positive correlation was found which confirms existing findings of Furnham and Winceslaus [12] and Gong and Furnham [15]. One surprising finding was that people familiar with personal psychiatric treatment recommend it less. This contradicts most other studies and the overall consensus within that field. However, we have no way of knowing why this result occurred as we have no details on the length and type of treatment that certain participants received or for what problem. This area certainly merits further investigation.

Like all studies in this area it had limitations, which mainly concerned the sample and the measure. It is always desirable to have a large representative sample of the population. The sample in this study was relatively big and varied enough to test the hypotheses but was overrepresented by younger and better educated people. It may be expected that the MHL of the general population would be lower. It would also be desirable, as done in some studies to have BPD patients, their relatives and those who specialize in treating them to examine systematic differences.

The second issue concerns the questionnaires and particularly the vignettes. Sai and Furnham [28] showed that different vignettes supposedly describing equally prototypically with OCPD were differentially recognize. This study had six BPD vignettes which showed that for most participants it was easier to detect BPD when comorbid with bipolar disorder, and least easy for NPD which they also found difficulty identifying and which supports previous work. However, we did not have a “pure” BPD vignette which would have been desirable. Further the “vignette” effect can be seen in Tables 1, 4 and 5 and which indicate that seemingly “equivalent” vignettes can produce very different results.

**Table 4:** Rates of BPD “Correct” identification in order of presentation

Mental disorder	Correct response (%)
BPD 1	4.1^a^
BPD 2	4.1^a^
BPD 3	0.5^a^
BPD 4	2.1^a^
BPD 5	1.0^a^
BPD 6	2.1^a^
Depression	72.5^b^
Schizophrenia	65.8^b^

Percentages that share the same superscript (a, b) are not significantly different from each other (*p* > 0.05)

*BPD* borderline personality disorder

**Table 5:** Identification rates of the comorbid disorders in the BPD Vignettes

Comorbid disorder	Response (%)
BP-II 1	5.7^a^
BP-II 2	1.6^a^
PTSD 1	15.0
PTSD 2	1.6^a^
NPD	1.0^a^
ASPD/substance abuse	28.5

Superscripted percentages (a) are not significantly different from each other (*p* > 0.05)

*BP-II* bipolar II disorder, *PTSD* post-traumatic stress disorder, *NPD* narcissistic personality disorder; *ASPD* antisocial personality disorder

Ignorance about BPD has important implications for MHL and clinical practice. It is possible that because BPD is not recognized as a mental disorder people receive castigation and ostracism rather than help. It is also possible that people with BPD are less likely to self-diagnose and seek help. Clearly greater knowledge of BPD would benefit those who have the disorder as well as their relatives and work colleagues who could offer help early once symptoms were spotted and a good diagnosis made.

## Appendix: How each vignette was described and rated

Mr. A, a 23-year-old man with divorced parents, developed an intense, idealized relationship with his very supportive but inexperienced substance abuse counselor. Because of Mr. A’s continuing to steal from his family and from stores and to drive too fast despite repeated encounters with the law, his mother sought consultation. When a change to a more confrontational and intensive therapy was recommended, Mr. A became very abusive and threatened his mother and stepfather with a knife. When his counselor, frightened by Mr. A’s desperate calls and by his threats to kill himself, joined the mother in support of a change in treatment, Mr. A ran away. The next contact from him was a telephone call apologizing for his flight and requesting his mother send him money to pay a debt and transport him home.
